# Effects of dietary L-glutamate and L-aspartate supplementation on growth performance, severity of diarrhea, intestinal barrier integrity, and fecal microbiota of weaned piglets challenged with F18 enterotoxigenic *Escherichia coli*

**DOI:** 10.1186/s40104-025-01266-x

**Published:** 2025-09-30

**Authors:** Supatirada Wongchanla, Kunal Dixit, Sangwoo Park, Kwangwook Kim, Shuhan Sun, Maria Marco, Steven B. Palomares, Alejandra Mejia-Caballero, Sahana Mohan, Xunde Li, Xiaojing Li, Yanhong Liu

**Affiliations:** 1 Department of Animal Science, University of California, Davis, USA; 2 Department of Food Science and Technology, University of California, Davis, USA; 3 Department of Animal Science, Michigan State University, East Lansing, USA; 4 Department of Population Health & Reproduction, University of California, Davis, USA; 5 United Animal Health, Sheridan, IN USA

**Keywords:** Diarrhea, *Escherichia coli*, Fecal microbiota, L-Aspartate, L-Glutamate, Weaned pigs

## Abstract

**Background:**

L-Glutamate and L-aspartate are functional amino acids that play pivotal roles in the cellular metabolic pathways of swine enterocytes. Therefore, this study aimed to investigate the effects of dietary L-glutamate and L-aspartate on growth performance, diarrhea severity, intestinal barrier integrity, and fecal microbiota of weaned piglets challenged with F18 enterotoxigenic *Escherichia coli* (ETEC). Weaned piglets were randomly assigned to seven dietary treatments, including unchallenged and ETEC-challenged controls, amino acid-supplemented groups, and an antibiotic control, to assess their responses to ETEC challenge.

**Results:**

Supplementation with 1% L-glutamate or 2% L-aspartate enhanced growth performance, with significantly greater (*P* < 0.05) average daily weight gain and gain-to-feed ratio compared with the positive control group from d 0 to d 5 post-inoculation. Pigs fed with 1% or 2% L-aspartate had reduced (*P* < 0.05) diarrhea severity in ETEC-challenged pigs compared with the positive control group. The 1% L-aspartate supplementation also supported intestinal structure by increasing (*P* < 0.05) duodenal villi height and ileal villi width compared with carbadox supplementation. Additionally, 1% L-glutamate supplementation significantly improved (*P* < 0.05) resilience in ETEC-challenged pigs by reducing fecal shedding of β-hemolysin-producing bacteria compared with the positive control group on d 14 post-inoculation. Moreover, 1% L-aspartate supplementation promoted intestinal barrier integrity by significantly up-regulated (*P* < 0.05) the expression of ileal *OCDN* and ileal *ZO-1* compared with the positive control group on d 14 post-inoculation. Interestingly, 2% L-aspartate supplementation altered the intestinal mucosa by down-regulating (*P* < 0.05) the expression of jejunal *CLDN-1*, while up-regulating (*P* < 0.05) the expression of ileal *CLDN-1* compared with the negative control group on d 14 post-inoculation. Furthermore, L-glutamate supplementation significantly changed proportions of Firmicutes and Bacteroidota and showed the trend for enrichment in beneficial bacterial genera such as *Bifidobacterium* and *Megasphaera* in ETEC-infected pigs by d 14 post-inoculation.

**Conclusion:**

Supplementation with L-glutamate or L-aspartate promoted growth performance, supported gut health, and enhanced disease resistance in weaned pigs challenged with F18 ETEC. During the weaning period, L-glutamate or L-aspartate could potentially be considered conditionally essential amino acids, helping to alleviate weaning complications and reduce the need for antibiotic use in swine farming.

**Supplementary Information:**

The online version contains supplementary material available at 10.1186/s40104-025-01266-x.

## Background

The weaning period presents significant challenges for piglets as they are abruptly introduced to various stressors. These biological and psychological stressors profoundly affect gut health and overall well-being by disrupting intestinal physiology, morphology, immunity, and microbiota balance [1]. A direct consequence of this stressful transition is underfeeding, which leads to gut disorders, impaired nutrient utilization, and ultimately growth retardation [2]. Post-weaning pigs are particularly susceptible to microbial invasion due to the lack of immunoglobulin-rich sow's milk and their immature intestinal immunity [3]. This vulnerability, along with the rise in serum cortisol levels caused by weaning stress, can compromise intestinal integrity and heighten epithelial permeability, making them more vulnerable to gut infection and inflammation [4–6]. As the gut microbiota has variety of roles including nutrient metabolism, immune regulation and protection from pathogen invasion in mammals, alterations in the microbiota during weaning is recognized as one of the important causes of post-weaning diarrhea (PWD) in pigs [7, 8]. These weaning complications result in decreased productive performance and substantial financial losses on a global scale. To mitigate these challenges and prevent infectious outbreaks, swine farms commonly rely on antibiotics or inorganic compounds such as high dose zinc oxide. However, the extensive use of antibiotics in livestock has contributed to the emergence of antimicrobial-resistant (AMR) bacteria, raising significant public health concerns [9]. Consequently, there is a crucial need to identify alternative strategies to address the challenges associated with weaning stress and AMR. Strategies such as housing control, biosecurity, immunization, genetic selection, and nutritional interventions are increasingly recognized for their crucial roles in improving both the health and overall performance of swine.

L-Glutamate (Glu) and L-aspartate (Asp) are functional amino acids that play critical roles in the metabolism of mammalian enterocytes [10]. They serve as major sources of energy for intestinal cells, providing adenosine triphosphate (ATP) and supporting cellular processes such as amino acid synthesis [11]. Previous studies have shown that dietary supplementation of Glu or Asp improved growth performance, alleviated oxidative stress, enhanced intestinal barrier integrity, and modulated gut immune functions in weaned piglets [12–14]. These amino acids have also been studied for their impact on the pig gut microbiome. Li et al. [15] showed that the inclusion of Asp in the diet of weaning pigs is correlated in changes in bacterial diversity, along with an increase in proportions of Actinobacteria. Moreover, both Glu and Asp have been found to reduce Firmicutes abundance in the pig intestine [16]. Given the combination of underfeeding experienced during the weaning process and the characteristics of modern fast-growing pig breeds, it becomes evident that there could be an increased necessity for Glu and Asp to adequately sustain their developmental needs during weaning stress condition [13].

PWD is a well-known challenge in swine production, leading to substantial economic losses worldwide. This condition typically results from intestinal colonization by enterotoxigenic *Escherichia coli* (ETEC) and the subsequent secretion of enterotoxins following weaning, leading to diarrhea [17, 18]. In the event of a PWD outbreak, certain large-scale pig farms have reported that up to 50% of piglets are affected by diarrhea, with mortality rates between 15% and 20%, which is noticeably worse in early-weaning piglets [19]. Although numerous studies have examined the roles of Glu and Asp in promoting gastrointestinal health and productivity in weaned pigs [14, 20–22], their specific effects on intestinal barrier integrity and the gut microbiome, particularly under pathogenic challenges such as ETEC infection, remain largely unexplored in the context of mitigating PWD. Thus, the primary objective of this study was to investigate the effects of dietary Glu and Asp supplementation on intestinal barrier integrity, gut microbiome, and overall health in weaned pigs challenged with ETEC. The study hypothesized that dietary Glu and Asp supplementation at both low (1%) and high (2%) doses would improve growth efficiency, mitigate diarrhea, and enhance intestinal integrity in weaned pigs challenged with ETEC. The low dose was selected to assess whether it is sufficient to achieve beneficial effects, while the high dose was included to determine if a higher level provides superior outcomes, allowing for the identification of the optimal dose for these effects. Additionally, the hypothesis proposed that Glu and Asp alters the fecal microbiota composition and diversity in weaned pigs, particularly under the stress of ETEC challenge, contributing to diarrhea alleviation.

## Materials and methods

### Animals, housing, experimental design, and diet

The protocol for this experiment was reviewed and approved by the Institutional Animal Care and Use Committee (IACUC #21875) of the University of California, Davis (UC Davis, USA). A total of 49 weanling pigs [24 d old; 8.18 ± 1.54 kg body weight (BW)] were used in this experiment. The 6 sows and piglets used in this experiment were not given *E. coli* vaccinations, antibiotic injections, or antibiotics in creep feed. All piglets were genotyped for susceptibility to F18 ETEC infection based on the *FUT1* gene polymorphism, using a polymerase chain reaction-restriction fragment length polymorphism (PCR-RFLP) assay. Briefly, genomic DNA was extracted from tail samples, and a specific region of the *FUT1* gene (Forward primer, 5′-CTT AGA CCT GCT GGC CCT GT-3′ and Reverse primer, 5′-AGG ACA GGC AGC GTG ATG-3′) was amplified by PCR. The PCR product was then digested with a restriction enzyme, HinP1I, that targets the polymorphic site. Piglets displayed two distinct DNA bands after digestion were identified as homozygous susceptible to F18 ETEC infection and were selected for this study. All piglets utilized in this experiment had not previously been infected with F18 ETEC. After weaning, pigs were housed in individual pens (0.61 m × 1.22 m) at the Cole facility at UC Davis. Pigs were contained in individual pens for 21 d, including a 7-d adaptation period and 14 d following the first ETEC inoculation. All pigs were given free access to diet and water. Animal units were provided with ventilators and heaters to create the ideal thermoneutral zone for nursery piglets. Light was provided for 12 h a day from 07:30 to 19:30.

Piglets were randomly allocated to one of seven treatments, with seven piglets per treatment, using a randomized complete block design with weight and sex as blocks and the individual pig as the experimental unit. The treatments included: 1) negative control (NC), basal diet without ETEC challenge, 2) positive control (PC), basal diet with ETEC challenge, 3) basal diet supplemented with 1% Glu with ETEC challenge, 4) basal diet supplemented with 2% Glu with ETEC challenge, 5) basal diet supplemented with 1% Asp with ETEC challenge, 6) basal diet supplemented with 2% Asp with ETEC challenge, and 7) antibiotic growth promotor (Car), basal diet supplemented with carbadox at 50 mg/kg and with ETEC challenge. The amino acid inclusion levels were selected based on estimations from a previous study [13] showing that, during the weaning period, dietary amino acids may become limiting due to a transient reduction in feed intake. The low inclusion level (1%) was intended to meet estimated nutritional requirements under weaning stress, while the high inclusion level (2%) was used to assess whether greater supplementation would elicit enhanced physiological responses or reveal a dose-dependent effect. These levels were chosen to help identify the optimal dosage for improving growth performance and mitigating weaning-associated challenges in piglets.

All diets met the current estimates for nursery pig nutrient requirements (Table 1 and Table S1) [23]. Diets did not include spray-dried plasma, antibiotics, or zinc oxide. All pigs were fed the experimental diets in a 2-phase feeding program, with weeks 1 and 2 serving as phase 1 and week 3 as phase 2.

**Table 1:** Ingredient compositions of experimental diets (as-fed basis)^1^

Ingredient, %	Control, phase I	Control, phase II
Corn	44.70	54.29
Dried whey	15.00	10.00
Soybean meal	21.50	30.50
Fish meal	3.00	-
Lactose	6.00	-
Soy protein concentrate	5.00	-
Soybean oil	2.00	2.00
Limestone	0.98	1.00
Dicalcium phosphate	0.55	0.90
L-Lysine·HCl	0.34	0.39
DL-Methionine	0.14	0.12
L-Threonine	0.09	0.10
Salt	0.40	0.40
Vit-mineral premix^2^	0.30	0.30
Total	100.00	100.00
Analyzed nutrient, % as-is basis		
Dry matter	91.30	89.70
Crude protein	21.55	21.17
Acid detergent fiber	3.47	4.31
Neutral detergent fiber	7.94	9.06

^1^In each phase, five additional diets were formulated accordingly

^2^Provided by the United Animal Health (Sheridan, IN, USA). Provided the following quantities of vitamins and micro minerals per kilogram of complete diet: Vitamin A as retinyl acetate, 11,136 IU; vitamin D_3_ as cholecalciferol, 2,208 IU; vitamin E as DL-alpha tocopheryl acetate, 66 IU; vitamin K as menadione dimethylprimidinol bisulfite, 1.42 mg; thiamin as thiamine mononitrate, 0.24 mg; riboflavin, 6.59 mg; pyridoxine as pyridoxine hydrochloride, 0.24 mg; vitamin B_12_, 0.03 mg; D-pantothenic acid as D-calcium pantothenate, 23.5 mg; niacin, 44.1 mg; folic acid, 1.59 mg; biotin, 0.44 mg; Cu, 20 mg as copper sulfate and copper chloride; Fe, 126 mg as ferrous sulfate; I, 1.26 mg as ethylenediamine dihydriodide; Mn, 60.2 mg as manganese sulfate; Se, 0.3 mg as sodium selenite and selenium yeast; and Zn, 125.1 mg as zinc sulfate

All pigs in the ETEC challenge treatments were orally inoculated with F18 ETEC for 3 consecutive days from d 0 before inoculation to d 2 post-inoculation (PI). The F18 ETEC was originally isolated from a field disease outbreak by the University of Montreal (isolate number: ECL22131). The F18 ETEC was given 10^10^ CFU per 3-mL dosage in phosphate-buffered saline (PBS), and it produces heat-labile toxin, and heat-stable toxin a and b. Our previous study [24, 25] indicated that this dose causes mild diarrhea.

### Diarrhea and clinical observations

Clinical observations (alertness and diarrhea scores) were recorded twice daily throughout the experiment, beginning on the weaning day (d −7). The alertness score of each pig was subjectively scored by two independent observers using a scale of 1 to 3 (1 = normal, 2 = slightly depressed or listless, and 3 = severely depressed or recumbent). Throughout the experiment, all pigs scored 1 on the alertness score (data not shown). The diarrhea score was subjectively scored by two independent observers, on a scale of 1 to 5 (1 = normal feces, 2 = moist feces, 3 = mild diarrhea, 4 = severe diarrhea, and 5 = watery diarrhea). The frequency of diarrhea (%) was calculated by the formula: [(pig days with diarrhea score of 3 or higher)/ (total pig days)] × 100 [25]. The frequency of severe diarrhea (%) was also calculated using the same formula as the pig days with diarrhea score of 4 or higher.

### Growth performance

Pigs and feeders were weighed on the day at weaning (d −7), d 0 before inoculation, d 5, and 14 PI. Average daily gain (ADG), average daily feed intake (ADFI), and gain-to-feed (G:F) ratio was calculated for each interval from d −7 to d 0, d 0 to d 5 PI, d −7 to d 5 PI, d 5 to d 14 PI and d −7 to d 14 PI for all pigs.

### Sample collections

The experiment terminated with the euthanasia of all pigs on d 14 PI. Pigs were anesthetized intramuscularly with a 1 mL mixture of 100 mg Telazol, 50 mg ketamine, and 50 mg xylazine (2:1:1). Following anesthesia, each pig was euthanized with an intracardiac injection of 78 mg Fatal-Plus solution (sodium pentobarbital, MWI Animal Health, Visalia, CA, USA) per 1 kg of body weight. The duodenum, middle of the jejunum, and ileum (5 cm close to the ileocecal junction) were collected in lengths of 4 cm per segment and fixed in 10% neutral buffered formalin for histological examination. Approximately 10 cm of the intestine was dissected longitudinally and gently washed with PBS to remove luminal contents for intestinal mucosa collection. Mucosa from the middle of the jejunum and ileum was scraped with a glass slide, promptly frozen in liquid nitrogen, and stored for gene expression analysis. Fecal samples from all pigs were taken directly from the rectum using cotton swabs on d 0 before inoculation, d 2, d 5, d 10, and d 14 PI for the detection of β-hemolytic coliforms [25]. Fecal samples were also collected on weaning day, d 0 before inoculation, and d 14 to perform 16S rRNA gene amplicon DNA sequencing.

### Detection of β-hemolytic coliforms

Feces from all pigs were cultured in Colombia Blood Agars with 5% sheep blood to detect hemolytic coliforms, as well as on MacConkey agars to confirm the presence of *E. coli* colonies. All plates were incubated at 37 °C for 24 h. Colonies of total coliforms and β-hemolytic coliforms on blood agars were visually examined. β-Hemolytic coliforms were scored from 0 to 8 based on the presence of the clear zone of hemolysis on the plate (0 = no bacterial growth, 8 = heavy bacterial growth). The ratio of β-hemolytic coliforms to total coliforms was calculated and reported as a percentage [25]. Questionable colonies were sub-cultured to determine if they were β-hemolytic *E. coli* using triple sugar iron agar and lysine iron agar, and to confirm the presence of the F18 fimbriae gene in *E. coli* using polymerase chain reaction (PCR) analysis [26].

### Intestinal morphology

The fixed intestinal segments were embedded in paraffin, sectioned at 5 μm, and stained with Hematoxylin and Eosin (H&E) staining method. The slides were photographed by an Olympus BX51 microscope at 10× and all measurements were conducted in the image processing and analysis software (Image J, NIH). Ten straight and integrated villi and their associated crypts and surrounded area were selected to analyze villus height, crypt depth, and villus height-to-crypt depth ratio (VCR).

### Intestinal barrier integrity

Reverse transcription quantitative PCR (RT-qPCR) was employed to assess gene expression in jejunal and ileal mucosa samples. Approximately 100 mg of each mucosa sample was homogenized with TRIzol reagent (Invitrogen; Thermo Fisher Scientific, Inc., Waltham, MA, USA). Subsequently, total ribonucleic acid (RNA) was extracted following the RNA extraction protocol recommendations provided by the reagent manufacturer. The High-Capacity Complementary Deoxyribonucleic acid (cDNA) Reverse Transcription Kit (Applied Biosystems; Thermo Fisher Scientific, Inc., Waltham, MA, USA) was used to generate cDNA from 1 μg of total RNA per sample in a 20-μL volume. To ensure RNA purity, absorbance ratios at 260 and 280 nm were measured with a Thermo Scientific NanoDrop 2000 Spectrophotometer (Thermo Scientific, Inc., Waltham, MA, USA). The mRNA expression levels of genes related to intestinal barrier integrity in jejunal and ileal mucosa were measured by RT-qPCR, including mucin-2 (*MUC-2*), zonula occludens-1 (*ZO-1*), claudin-1 (*CLDN-1*), and occludin (*OCDN*). Data normalization was achieved using 18S ribosomal RNA (*18S rRNA*) as a housekeeping gene. Primers were designed according to published literature and commercially produced by Integrated DNA Technologies in Coralville, IA, USA. All primers underwent thorough verification and optimization before being analyzed for RT-qPCR (Table S2). A no template control (NTC) was included as a negative control to assess reagent contamination and the potential presence of genomic DNA. The analysis of relative gene quantification in comparison to the negative control was conducted using the 2^−ΔΔCT^ method. The RT-qPCR reaction conditions adhered to the protocols outlined in the published research [24].

### Fecal microbiota profiling and data analysis

A total of 30, 49 and 49 fecal samples were collected on the weaning day (d −7), before ETEC inoculation (d 0), and on the final day of the experiment (d 14 PI), respectively, and stored at −80 °C prior to further analysis. DNA extraction was performed using the DNeasy PowerSoil Pro Kit (Cat. No. 47016, Qiagen, USA) as per the manufacturer’s instructions with the exception that a bead beating step using FastPrep-24 5G bead beater (MP Biomedicals, USA) at a speed of 6.5 m/s for 60 s as described previously [27]. DNA concentration and purity were determined on a NanoDrop 2000c Spectrophotometer (NanoDrop Biotechnologies, USA). The polymerase chain reaction (PCR) was performed using a barcoded forward F515 (5′-GTG TGC CAG CMG CCG CGG TAA-3′) primer and reverse primer R806 (5′-GGA CTA CHV GGG TWT CTA AT-3′) (Integrated DNA Technologies, USA) to obtained amplified V4 regions of 16S rRNA [28]. Twenty-five cycles of PCR were run with initial denaturation step of 94 °C for 3 min followed by 25 cycles of denaturation (94 °C, 45 s), annealing (50 °C, 60 s), extension (72 °C, 30 s) and final extension at 72 °C for 10 min. PCR amplicons were tested for quality and quantity using agarose gel electrophoresis and Qubit 4 (Fisher Scientific, USA), respectively, and then diluted to 40 ng/µL. Pooled amplicons were purified using the Wizard SV Gel and PCR Clean-Up System (Promega, USA) prior to submission to the University of California, Davis, Genome Center for sequencing on the AVITI sequencing platform (Element Biosciences, San Diego, USA) with 2 × 300 medium output sequencing chemistry (https://dnatech.genomecenter.ucdavis.edu/). Revvity NEXTFLEX Unique Dual Index Barcodes (8NT index, 1–96) were used for the sequencing library preparation along with Truseq-style sequencing adapters. The sequence data are available in the Bioproject PRJNA1184791.

A total of 209,033 ± 89,680 reads were obtained per sample after sequencing. Raw data were demultiplexed with flexbar tool (v 3.5.0) prior to downstream analysis with the open source DADA2 (v1.16) pipeline available at http://benjjneb.github.io/dada2/tutorial_1_6.html [29, 30]. Initial primer removal was performed using the trimLeft parameter during data filtering. Sequence reads below a Phread quality score of 30 (Q30) and read length < 250 bp for R1 reads and < 260 bp for R2 reads were filtered out. To reduce bias from uneven depth, all samples were normalized after DADA2 processing to 39,373 reads/sample based on rarefaction curve assessment. Taxonomy was assigned to amplicon sequence variants (ASVs) using the SILVA (v 138) database after read denoising, merging and removal of chimeric sequences [31]. Data were further analysed using R (v 4.2.2). Alpha diversity indices, Observed ASVs, Shannon index and Simpson’s diversity index, along with beta diversity assessed by principal coordinate analysis (PCoA) were calculated to determine overall microbiota differences between study groups (*P*_*adj*_ < 0.05, Wilcoxon signed-rank test and Permutational Multivariate Analysis of Variance respectively). Adonis2 from vegan package and PCoA using weighted UniFrac distances were employed to determine significance between study groups. Differentially abundant taxa between the treatment groups were calculated to determine the effect of dietary supplementation over the period of study. Significant changes in microbial community structure between the study groups were determined using DESeq2 likelihood ratio test (LRT) analysis with Benjamini–Hochberg method to calculate adjusted *P*-values.

### Statistical analysis

Data normality was assessed utilizing the UNIVARIATE procedure in SAS. All data, excluding the frequency of diarrhea and fecal microbiota, were analyzed using the Proc Mixed of SAS (SAS Inst. Inc, Cary, NC, USA). The statistical model used treatment as a fixed effect, blocks (sex and body weight) as random effects, and individual pig as the experimental unit. Treatment means were computed utilizing the LSMEANS statement, and means were separated using the PDIFF statement and adjusted with Tukey. The Chi-square test was applied to assess the frequency of diarrhea. Statistical significance and tendency were defined as *P* ≤ 0.05 and 0.05 < *P* ≤ 0.10, respectively.

## Results

### Glu and Asp supplementation on growth performance and diarrhea frequency

Pigs supplemented with 1% Glu, 2% Asp, or carbadox showed significant improvements (*P* < 0.05) in ADG and G:F ratio compared with pigs in the PC during d 0 to d 5 PI, the peak diarrhea period (Table 2). Among these, supplementation with 1% Glu resulted in a higher (*P* < 0.05) ADG compared with 1% Asp during the same period. Interestingly, pigs fed 2% Asp showed ADFI levels comparable to the NC but had higher (*P* < 0.05) ADFI than those supplemented with 2% Glu during d −7 to d 0, the first week after weaning. However, no differences in BW were observed across all treatments at any time point throughout the experiment.

**Table 2:** Growth performance of ETEC-challenged weaned pigs fed experimental diets

Item^1^	NC	PC	1% Glu	2% Glu	1% Asp	2% Asp	Carbadox	SEM	*P*-value
BW, kg									
d −7	8.16	8.17	8.09	8.19	8.38	8.20	8.08	0.62	1.00
d 0	9.38	8.99	9.28	9.16	9.43	9.40	9.24	0.67	1.00
d 5 PI	9.91	9.55	10.26	9.57	9.69	10.35	10.22	0.80	0.95
d 14 PI	14.57	14.47	15.67	14.50	15.17	15.63	15.13	1.16	0.95
ADG, g									
d −7 to 0	172.87	115.93	171.01	136.95	148.58	170.62	164.09	27.32	0.51
d 0 to 5 PI	175.34^abc^	37.65^c^	250.87^a^	136.14^abc^	96.48^bc^	228.49^ab^	193.92^ab^	54.87	0.09
d 5 to 14 PI	517.62	546.67	601.90	548.41	637.31	586.03	545.56	49.27	0.61
ADFI, g									
d −7 to 0	410.53^a^	322.77^ab^	369.07^ab^	284.00^b^	357.46^ab^	410.53^a^	369.71^ab^	34.83	0.08
d 0 to 5 PI	383.33	337.33	499.67	373.33	421.33	528.67	438.29	50.08	0.12
d 5 to 14 PI	868.25	767.62	865.24	782.86	862.38	958.73	875.08	84.77	0.65
G:F ratio									
d −7 to 0	0.41	0.35	0.46	0.49	0.42	0.45	0.48	0.05	0.55
d 0 to 5 PI	0.46^a^	−0.05^b^	0.51^a^	0.31^ab^	0.15^ab^	0.45^a^	0.41^a^	0.16	0.10
d 5 to 14 PI	0.60^c^	0.74^a^	0.71^ab^	0.71^abc^	0.72^ab^	0.63^bc^	0.62^bc^	0.04	0.06

^1^*BW *Body weight, *ADG *Average daily gain, *ADFI *Average daily feed intake, *G:F *Gain-to-feed, *PI *Post-inoculation. Each least squares mean represents 7 replicates (*n* = 7 pigs per treatment). ^a−c^Means without a common superscript are different (*P* < 0.05)

**Fig. 1:**
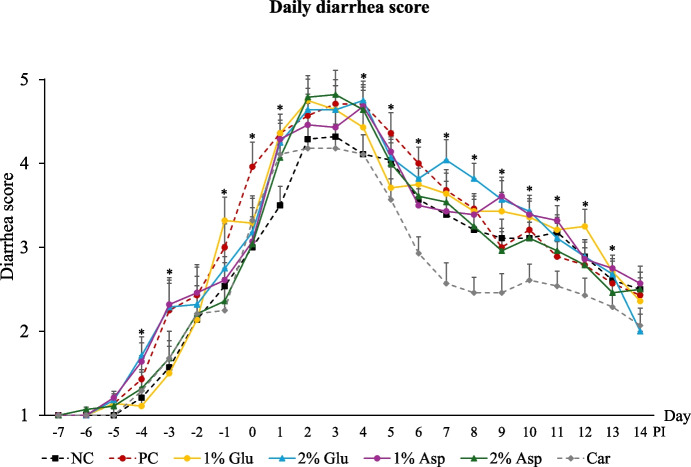
Daily diarrhea score throughout the experiment. Daily diarrhea score of weaned pigs fed diets supplemented with glutamate (Glu), aspartate (Asp), or carbadox (Car). Diarrhea score = 1, normal feces, 2, moist feces, 3, mild diarrhea, 4, severe diarrhea, 5, watery diarrhea. NC = negative control; PC = positive control; PI = post-inoculation. Each least squares mean represents 7 replicates (*n* = 7 pigs per treatment). *P < 0.05, indicating fecal scores were different among treatments

**Fig. 2:**
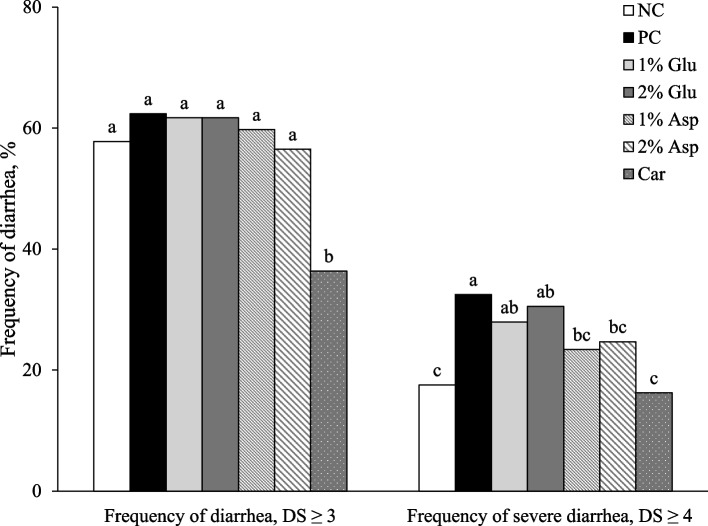
Frequency of diarrhea throughout the experiment. Frequency of diarrhea of weaned pigs fed diets supplemented with glutamate (Glu), aspartate (Asp), or carbadox (Car). Frequency of diarrhea was calculated as the percentage of pig days with diarrhea score ≥ 3 in the total of pig days. Frequency of severe diarrhea was calculated as the percentage of pig days with diarrhea score ≥ 4 in the total of pig days. NC = negative control; PC = positive control; DS = diarrhea score. ^a−c^Means without a common superscript are different (*P* < 0.05)

Diarrhea was induced by the oral challenge with F18 ETEC. Fecal scores differed among treatments (*P* < 0.05) from d 4 to d 13 PI, with pigs supplemented with carbadox generally showing lower scores than others (Fig. 1). Notably, during certain periods, no differences in daily diarrhea scores were observed between carbadox and 2% Asp supplementation, specifically from d −7 to d 5 PI and d 9 to d 14 PI, indicating that 2% Asp supplementation had effects similar to those of the carbadox group. Similarly, no significant differences in daily diarrhea scores were detected among the NC, 1% Glu, 1% Asp, and 2% Asp groups from d 2 to d 14 PI. Interestingly, pigs supplemented with 2% Glu showed daily diarrhea scores comparable to those in the PC throughout d 1 to d 14 PI. In terms of the frequency of diarrhea (diarrhea score ≥ 3), pigs fed with carbadox showed the lowest (*P* < 0.05) values over the entire experiment (Fig. 2). Furthermore, compared with the PC, pigs in the NC, 1% Asp, 2% Asp, and carbadox groups had lower (*P* < 0.05) frequency of severe diarrhea (diarrhea score ≥ 4) (32.47% vs. 16.23% to 24.68%). However, no significant differences in the frequency of severe diarrhea were observed among the PC, 1% Glu, and 2% Glu groups.

### Glu and Asp supplementation on fecal β-hemolytic coliforms

**Fig. 3:**
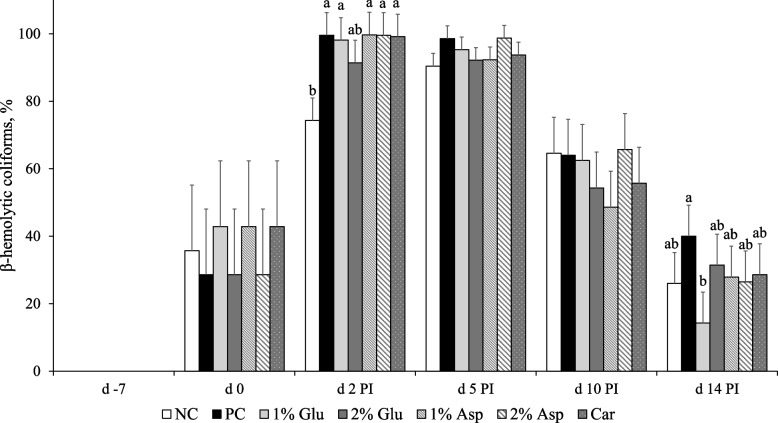
The percentage of β-hemolytic coliforms throughout the experiment. The percentage (%) of β-hemolytic coliforms in total coliforms in fecal samples of weaned pigs fed diets supplemented with glutamate (Glu), aspartate (Asp), or carbadox (Car). NC = negative control; PC = positive control; PI = post-inoculation. Each least squares mean represents 7 replicates (*n* = 7 pigs per treatment). *P < 0.05, indicating the percentage of β-hemolytic coliforms in total coliforms in fecal samples were different among treatments. ^a,b^Means without a common superscript are different (*P* < 0.05)

No differences were observed in the percentage of fecal β-hemolytic coliforms relative to total coliforms among all treatments on d 0, prior to F18 ETEC inoculation. Following ETEC challenge, β-hemolytic coliforms were detected in the feces of all pigs on d 2 PI (Fig. 3). At this time point, pigs in the PC, 1% Glu, 1% Asp, 2% Asp, and carbadox groups exhibited a higher (*P* < 0.05) percentage of fecal β-hemolytic coliforms compared with those in the NC group. No significant differences in fecal β-hemolytic coliform percentages were observed among the treatments on d 5 and d 10 PI. At d 14 PI, pigs supplemented with 1% Glu showed a significantly lower (*P* < 0.05) percentage of fecal β-hemolytic coliforms compared to those in the PC group.

### Glu and Asp supplementation on intestinal morphology across different segments

At necropsy (d 14 PI), notable differences in intestinal morphology were observed among the treatments (Table 3). In the duodenum, pigs fed with 1% Asp showed a higher (*P* < 0.05) villi height compared with those in the NC, PC, 1% Glu, 2% Glu, or carbadox groups. In addition, pigs fed with carbadox showed the shortest (*P* < 0.05) crypt depth and the highest (*P* < 0.05) VCR among all treatments in the duodenum. Interestingly, in the jejunum, pigs fed with 1% Glu had the shortest (*P* < 0.05) crypt depth, whereas supplementation with 2% Glu had the longest (*P* < 0.05) crypt depth, indicating that supplementation with 1% Glu could help mitigate intestinal disturbances. In the ileum, pigs supplemented with 1% Glu, 2% Glu, or 1% Asp had greater (*P* < 0.05) villi width compared with pigs in the carbadox group. However, no significant differences were observed in villi height or VCR in the jejunum and ileum across treatments.

**Table 3:** Intestinal morphology of ETEC-challenged weaned pigs fed experimental diets on d 14 post-inoculation

Item^1^	NC	PC	1% Glu	2% Glu	1% Asp	2% Asp	Carbadox	SEM	*P*-value
Duodenum									
Villi height, μm	277.99^b^	274.03^b^	262.58^b^	286.48^b^	328.92^a^	293.97^ab^	267.09^b^	18.93	< 0.05
Villi width, μm	108.93	104.91	117.05	108.85	116.14	105.67	98.58	5.00	0.16
Crypt depth, μm	288.30^a^	251.89^ab^	283.28^a^	278.69^a^	291.31^a^	282.14^a^	224.49^b^	15.89	< 0.05
VCR	1.02^bc^	1.16^ab^	0.98^c^	1.07^abc^	1.19^ab^	1.12^abc^	1.21^a^	0.09	0.10
Jejunum									
Villi height, μm	318.70	331.17	324.60	319.20	328.49	295.68	293.81	20.75	0.48
Villi width, μm	74.55	81.30	80.52	77.28	76.55	76.47	75.48	4.32	0.71
Crypt depth, μm	186.66^abc^	181.89^abc^	159.19^c^	208.07^a^	185.47^abc^	197.91^ab^	176.49^bc^	12.03	0.09
VCR	1.91	1.95	2.16	1.70	1.96	1.62	1.84	0.19	0.34
Ileum									
Villi height, μm	223.97	223.92	234.51	229.78	235.13	229.08	201.29	9.72	0.25
Villi width, μm	73.87^ab^	74.03^ab^	81.06^a^	80.98^a^	76.85^a^	73.55^ab^	64.83^b^	3.70	0.05
Crypt depth, μm	164.66	164.43	170.26	167.66	169.62	188.52	155.12	12.86	0.71
VCR	1.47	1.34	1.54	1.56	1.48	1.31	1.42	0.13	0.72

^1^*VCR *Villi height-to-crypt depth ratio. Each least squares mean represents 7 replicates (*n* = 7 pigs per treatment). ^a−c^Means without a common superscript are different (*P* < 0.05)

### Glu and Asp supplementation on tight junction gene expression in intestinal mucosa

**Fig. 4:**
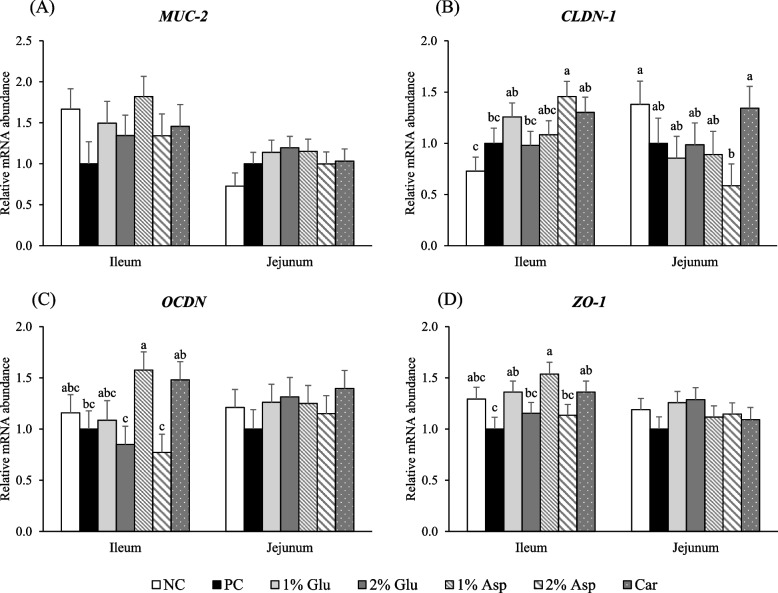
The mRNA expression of intestinal barrier integrity biomarkers on d 14 post-inoculation. Relative mRNA abundance of *MUC-2* (**A**), *CLDN-1* (**B**), *OCLN* (**C**), and *ZO-1* (**D**) in jejunal and ileal mucosa of weaned pigs fed diets supplemented with glutamate (Glu), aspartate (Asp), or carbadox (Car) on d 14 PI. NC = negative control; PC = positive control. *CLDN-1* = Claudin-1; *MUC-2* = Mucin-2; *OCLN* = Occludin; *ZO-1* = Zonula occludens-1. Each least squares mean represents 7 replicates (*n* = 7 pigs per treatment). ^a−c^Means without a common superscript are different (*P* < 0.05)

No difference was observed in transcript levels of *MUC-2* in both jejunal and ileal mucosa among all treatments (Fig. 4A). Pigs fed with 2% Asp showed significantly different (*P* < 0.05) *CLDN-1* transcript levels compared with the NC group in both intestinal regions (Fig. 4B). Specifically, *CLDN-1* expression was lower (*P* < 0.05) in the jejunum and higher (*P* < 0.05) in the ileum relative to NC. Although these differences were statistically significant, the relative abundances did not exceed a twofold change, suggesting a modest but region-specific modulation of tight junction gene expression by 2% Asp supplementation. Furthermore, pigs fed with 1% Asp had higher (*P* < 0.05) mRNA levels of *OCDN* and *ZO-1* in the ileal mucosa compared to pigs in the PC, 2% Glu, and 2% Asp groups (Fig. 4C and D). Pigs fed 1% Glu also had higher (*P* < 0.05) mRNA levels of *ZO-1* in the ileal mucosa compared with pigs in the PC (Fig. 4D). In addition, pigs fed 2% Glu or 2% Asp showed similar trends in expression of *OCDN* and *ZO-1* in ileal mucosa as those in the PC group. Moreover, pigs fed with 1% Glu or 1% Asp showed similar trends as pigs fed with carbadox in transcript quantities of *CLDN-1, OCDN,* and *ZO-1* in both jejunal and ileal mucosa. However, no difference was observed in mRNA levels of *OCDN* and *ZO-1* in jejunal mucosa among all treatments. Nevertheless, confirmation at the protein level is needed to validate these transcriptomic findings.

### Glu and Asp supplementation on fecal microbial diversity and taxonomic composition

Microbial composition was compared in pigs over time at different fecal collection time points (d −7, d 0, and d 14 PI), as well as across treatment groups. The bacterial contents of the pig fecal microbiota mainly consisted of Firmicutes and Bacteroidota, followed by Actinobacteria and Proteobacteria (Fig. S1A). The proportions of Firmicutes increased significantly on d 14 (76.23% ± 4.92%) compared to d 0 (65.91% ± 11.48%) (*P*_adj_ < 0.05, Wilcoxon test). An opposite trend was observed for bacteria in the Bacteroidota phylum [(23.64% ± 8.84%) on d 0; (18.77% ± 4.26%) on d 14]. Significantly higher proportions of Actinobacteria and Proteobacteria were reported on d 0 compared to other timepoints. In addition to changes in the major phyla, lower abundant phylum such as Euryarchaeota was found to decrease over time (Fig. S1B).

**Fig. 5:**
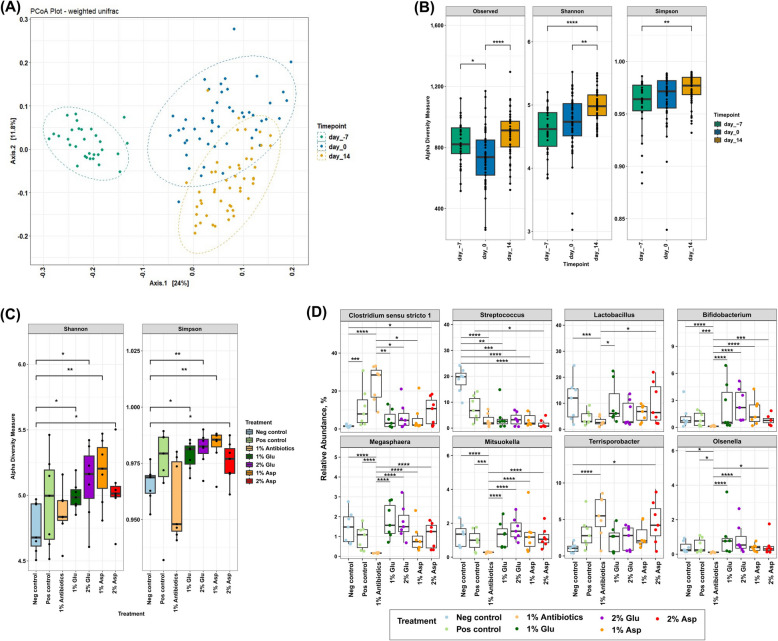
Pig fecal microbiota changes over time and with limited responses to antibiotic and amino acid dietary supplements. **A** Weighted UniFrac distance metric between three timepoints. **B** Alpha diversity measures comparing three timepoints. **C** Alpha diversity measures comparing seven treatment groups on d 14 post-inoculation. **D** Differentially abundant taxa on d 14 post-inoculation. Significant differences between the groups were marked based on the adjusted *P*-value (* < 0.05, ** < 0.01, *** < 0.005, **** < 0.001)

Bacterial beta-diversity analysis based on the weighted UniFrac metric showed that the fecal bacterial composition changed from the day of weaning to the post-weaning period (PERMANOVA, *P*_*adj*_ < 0.01) (Fig. 5A). Alpha-diversity measures, including Shannon and Simpson indices, indicated an increase in bacterial diversity in the fecal samples over time from d −7 to d 14 PI (*P*_*adj*_ < 0.01) (Fig. 5B). Fecal samples at d 0 had significantly lower (*P*_*adj*_ < 0.05) numbers of observed ASVs compared to those at d −7 and d 14 PI (Fig. 5B). Fecal microbial diversity among treatment groups at each timepoint showed limited differences on d −7 and d 0 (Fig. S2). However, at the end of the study (d 14 PI), the fecal microbiota from pigs supplemented with either Glu or Asp showed a significantly higher (*P*_*adj*_ < 0.05) alpha diversity indices compared to NC group (Fig. 5C).

Genus-level bacterial composition revealed that the pig fecal microbiota was dominated by *Prevotella, Lactobacillus, Agathobacter,* and *Clostridium sensu stricto *1 (Fig. S3). Overall, the taxonomic composition changed over time, but there were no significant differences in individual bacterial taxa proportions at either d −7 or d 0 across the treatment groups (*P*_*adj*_ > 0.05; LRT analysis DESeq2). By d 14 PI, 29 genera showed significantly different proportions (*P*_*adj*_ < 0.05) (Fig. 5D and Fig. S4). Bacteria in the genus *Streptococcus* were significantly higher (*P*_*adj*_ < 0.05) in the fecal contents of the healthy control group (NC) (18.50% ± 4.90%). *Clostridium sensu stricto *1 was present in significantly higher (*P*_*adj*_ < 0.05) proportions in pigs administered carbadox (23.69% ± 9.53%) compared to other groups (Fig. 5D). Conversely, *Megasphaera* and *Bifidobacterium* were found in lower proportions (*P* < 0.05) in carbadox-fed pigs (0.17% ± 0.02% and 0.13% ± 0.06%, respectively) compared to other groups; and tended to be higher in pigs fed with Glu, although the increase was not significant (Fig. 5D). Bacteria from the genera *Lactobacillus, Mitsuokella,* and *Olsenella* were also observed in smaller proportions (3% ± 1.68%, 0.28% ± 0.04%, and 0.11% ± 0.03%) in pigs supplemented with carbadox. The genus *Terrisprobacter* was enriched in the 2% Asp-fed pigs (4.57% ± 2.88%) compared with the NC group (1.06% ± 0.61%) (Fig. 5D).

## Discussion

Piglets experiencing weaning stress often encounter various physiological and psychological complications, which disrupts gut development, impairs immune functions, reduces feed consumption and nutrient utilization. These challenges ultimately hinder the ability of weaned piglets to achieve optimal growth and increase their susceptibility to microbial infections [32]. Glu and Asp are functional amino acids that play crucial roles in key metabolic pathways and cellular activities in mammalian cells [10]. While Glu and Asp are typically regarded as nutritionally non-essential amino acids, under conditions of weaning stress where piglets suffer inadequate feed intake, they may be considered conditionally essential to fulfill specific physiological requirements [33]. Despite their biological importance, limited information is available regarding the effects of dietary Glu and Asp on the growth performance and gut health of weaned piglets. In the present study, supplementation with Glu or Asp at selected dosages showed potential to alleviate growth suppression, reduce diarrhea severity, improve specific aspects of intestinal morphology and barrier-related gene expression, and modulate gut microbiota composition in weaned piglets challenged with F18 ETEC. These findings suggested that targeted amino acid supplementation may offer partial benefits comparable to antibiotic use, although further studies are needed to confirm functional outcomes at the protein level and over extended periods. The results were consistent with previous reports demonstrating the beneficial roles of Glu and Asp in promoting post-weaning recovery in pigs [12, 21, 22].

The deficiencies of multiple nutrients during weaning stress, including amino acids, contribute to the weaning associated growth depression syndrome [13]. In this study, supplementing with 1% Glu or 2% Asp showed promising results in improving ADG and G:F ratio of pigs infected with F18 ETEC during the first week post-infection, suggesting mitigation of growth retardation caused by the bacterial challenge. These improvements may be attributed to enhanced enterocyte metabolism and nutrient utilization, as Glu and Asp serve as key energy substrates for intestinal cells, supporting essential cellular functions during recovery [22, 34]. Given that intestinal cells require substantial energy to maintain their functions, energy deficiency can impair nutrient absorption and ultimately compromise piglet growth [35]. Consistent with previous findings, this study also showed that Glu and Asp improved ADFI and ADG in piglets under oxidative stress induced by hydrogen peroxide [12]. Previous studies have also reported that supplementation of Glu and Asp enhanced nutrient digestibility, thereby contributing to the improvement of growth performance in piglets [20, 22, 36]. Therefore, sufficient provision of Glu and Asp in the diet of nursery pigs may serve as intestinal fuels that support gut functions, improve nutrient utilization, and promote growth in weaned pigs. However, our findings indicated that 2% Glu reduced growth performance similarly to the PC group, contrasting with previous studies where higher Glu doses yielded better results [12, 37]. We suggested further investigating the potential amino acid burden associated with high doses of Glu by examining blood urea nitrogen (BUN) levels and the amino acid profile, as these factors could impact productivity and overall health of piglets.

Weaning stress disrupts the intestinal health and microenvironment of piglets making them more susceptible to pathogenic microorganisms such as ETEC, which, in turn, leads to PWD [32]. The present study clearly demonstrated a reduction in the severity of diarrhea through the supplementation of Asp to weaned piglets challenged with F18 ETEC. Furthermore, feeding 1% Glu to infected pigs reduced the shedding of β-hemolytic *E. coli* in their feces, which is an indirect indicator of F18 ETEC shedding, suggesting a faster recovery from bacterial infection during the infectious period. These results were closely in agreement with the findings from a previous study which reported that adequate and appropriate levels of amino acid provision can mitigate PWD [38].

Intestinal structure and function depend on the balance between intestinal epithelial cell proliferation and apoptosis. Weaning stress disrupts these processes, leading to villi atrophy and crypt hyperplasia, while ETEC infection further exacerbates intestinal morphology damage, ultimately impairing nutrient utilization and causing malnutrition in piglets [32]. In this study, supplementation with Glu and Asp was associated with changes in key morphometric parameters, including villus height, crypt depth, and villus width across different intestinal segments. The observed structural alterations may be partly explained by the potential modulation of enterocyte proliferation and survival, as Glu and Asp can serve as metabolic fuels and may influence the mammalian target of rapamycin (mTOR) signaling pathway, which is associated with maintaining intestinal integrity and supporting nutrient absorption [20, 39]. Additionally, Glu and Asp supplementation may increase mucosal levels of glutathione and ATP, helping protect intestinal cells from damage during the stressful weaning period [13, 39]. These findings suggested a potential modulatory role of Glu and Asp in intestinal development during the early post-weaning period. It is worth noting that amino acid supplementation may have region-specific effects along the gut, and the influence of higher inclusion levels on intestinal morphology warrants further investigation.

Epithelial tight junction proteins, such as claudin-1, occludin, and zonula occludens-1, along with mucins, are crucial for maintaining the integrity of the gut barrier. They help regulate paracellular permeability, protecting the host from luminal microorganisms, invading pathogens, and external toxins [40]. ETEC infection disrupts the assembly of tight junction proteins, leading to compromised intestinal barrier integrity. This disruption triggers an increase in pro-inflammatory cytokines, which further downregulates tight junction protein gene expression, exacerbating permeability [41]. Our study revealed that supplementation with 1% Glu resulted in tight junction protein expression levels in the ileal mucosa comparable to those observed in the carbadox group, suggesting a potential role in maintaining gut barrier function. Conversely, 2% Glu supplementation led to a reduction in the expression of tight junction genes in the ileal mucosa, a finding that contrasts with the beneficial effects observed at lower supplementation levels. Additionally, pigs supplemented with 1% Asp exhibited increased jejunal claudin-1 and occludin gene expression, similar to the carbadox group, supporting its possible positive effects on intestinal barrier integrity. Interestingly, supplementation with 2% Asp reduced claudin-1 expression in the jejunal mucosa while upregulating it in the ileum, indicating a dose- and tissue-specific effect. These results aligned with previous studies. For instance, Wang et al. [14] reported that pigs fed with 0.5% and 1% Asp increased jejunal claudin-1 and occludin protein expression after a 4 h of lipopolysaccharide (LPS) challenge, consistent with our findings of 1% Asp supplementation [14]. Nevertheless, Qin et al. [20] found that 2% Glu supplementation increased tight junction protein expression in weaned pigs challenged with LPS, which contrasted with our findings of reduced tight junction gene expression in the ileal mucosa of pigs supplemented with 2% Glu [20].

However, as this study assessed only mRNA expression, interpretations regarding barrier function should be made with caution. In the absence of corresponding protein expression data or functional assessments (e.g., intestinal permeability assays), it remains unclear whether the observed transcriptomic changes translate into alterations in epithelial barrier integrity. A further investigation into the potential effects of supplementing high doses of Glu and Asp in conjunction with ETEC infection on epithelial barrier function is suggested. Our observations were conducted during the late infection period (d 14 PI), and the observed effects may vary based on factors such as dosage, tissue type, or infection phase. Therefore, future studies should include protein-level analyses and time-course observations during the peak diarrhea phase to better understand the temporal dynamics and functional implications of these transcript-level changes. Collectively, while our findings highlight transcriptomic alterations in tight junction-related genes, additional validation is required to determine whether Glu and Asp supplementation can influence intestinal barrier function in F18 ETEC-challenged pigs. Moreover, it is important to note that the RT-qPCR analysis in this study utilized only a single housekeeping gene for normalization, although the use of multiple reference genes is recommended to improve data reliability.

Investigation of gut microbiota changes as a result of this nutrient supplementation is essential, as imbalance in microbial community structure may lead to intestinal inflammation [42]. Previously, weaning stress and nutrient supplementation have been shown to have an impact on microbiota structure [43–45]. In agreement with previous findings, the post-weaning transition significantly impacted both alpha- and beta-diversity of bacteria in pig stools, most likely due to the dietary transition. In addition to the notable effects of Glu and Asp on diarrhea and fecal shedding, supplementing with Glu or Asp facilitated subtle shifts in the gut microbiota, especially at the genus level. Dietary supplementation of Glu and Asp was previously found to regulate intestinal microbial composition by enhancing the abundance of short-chain fatty acids-producing bacteria [43]. Furthermore, Li et al. [15] and Liu et al. [16] also reported that 1% L-Asp supplementation increased alpha diversity and was associated with an increased abundance of *Lactobacillus*, *Prevotellaceae-NK3B31-group,* and *UCG-005* in the cecum of weaned pigs [15]. In the present study, pigs treated with antibiotics were found to have more changes in the fecal microbiota structure compared to other groups on d 14 PI. The low abundance of known commensal and beneficial taxa such as *Lactobacillus, Bifidobacterium* and *Olsenella,* along with possible probiotic taxa such as *Megasphaera* and *Mitsuokella,* were found in the fecal contents of pigs supplemented with antibiotics [43, 46]. This observation coupled with high abundance pro-inflammatory genera *Terrisporobacter* [43] and *Clostridium* *sensu stricto* 1 [47] could indicate the un-targeted nature of the antibiotics used.

Several potential mechanisms might contribute to the reduction of diarrhea, support gastrointestinal health, and improve growth performance in piglets supplemented with Glu and Asp, based on previous research and our current study. First, Glu and Asp can be catabolized in enterocytes to supply energy and generate other amino acids and nitrogenous substances, thereby supporting gut development and repairing intestinal damage [13, 22, 37]. Second, Glu and Asp exhibited beneficial effects by promoting antioxidant activities and alleviating oxidative stress in enterocytes, which supports mucosal barrier integrity [12, 37]. Third, Glu and Asp exerted intestinal benefits through their metabolites. Both amino acids can be converted into arginine, which helps maintain sufficient intracellular levels of arginine necessary for sustaining high nitric oxide (NO) production rates. NO, in turn, plays a crucial role in alleviating intestinal inflammation and mucosal injury by supporting intestinal blood circulation and contributing to normal physiological functioning of intestine [22, 34, 48]. Finally, Glu and Asp supplementation in weaned diets may influence microbiota characteristics, potentially mitigating dysbiosis commonly associated with PWD [38, 49, 50]. It is important to note that many of these mechanisms should be further investigated and verified in vivo to thoroughly understand the importance of Glu and Asp.

## Conclusions

In conclusion, dietary supplementation with selected doses of Glu or Asp mitigated growth suppression, alleviated severe diarrhea, supported intestinal integrity comparable to the antibiotic carbadox, and modulated the gut microbiota in weaned pigs infected with F18 ETEC. Both Glu and Asp are functional amino acids that not only contribute to protein synthesis but also address health issues and enhance productive performance in weaned piglets. Under the stress conditions of weaning, dietary Glu and Asp could potentially be classified as conditionally essential amino acids. Supplementing swine diets with Glu or Asp could reduce the need for antibiotics treatment and mitigate AMR by promoting gut health of weaned pigs. This study indicated that a low dose of dietary Glu and Asp, at 1% inclusion, was both sufficient and effective in nursery pig diets to improve piglet health and mitigate PWD. Future research is needed to investigate the effects of Glu and Asp on immune responses and metabolite profiles to uncover any direct influences these amino acids may exert. Additionally, investigating the combined effects of Glu and Asp could reveal further benefits in alleviating weaning stress complications.

## Supplementary Information

Supplementary Material 1. Table S1 Amino acid profiles of experimental diets. Table S2 Sequences of oligonucleotide primers used for RT-qPCR assay. Fig. S1 Bacterial phyla in pig gut. A Phylum level microbial distribution in pig gut. B Significant differences between phyla proportions were found at different timepoints. Fig. 2 Alpha diversity differences between different treatment groups on d −7 (A) and d 0 (B) are not significant. Fig. S3 Distribution of 20 most abundant genera in pigs in different treatment groups. Fig. 4 Significant differences in taxa proportions were found between treatment groups on d 14 post-inoculation.

## Data Availability

All data generated or analyzed during this study are available from the corresponding author upon reasonable request.

## Abbreviations

ADG: Average daily gain
ADFI: Average daily feed intake
AMR: Antimicrobial-resistant
Asp: L-Aspartate
ASV: Amplicon sequence variants
ATP: Adenosine triphosphate
BW: Body weight
BUN: Blood urea nitrogen
Car: Carbadox
CFU: Colony-forming unit
CLDN-1: Claudin-1
cDNA: Complementary deoxyribonucleic acid
ETEC: Enterotoxigenic *Escherichia coli*
EDTA: Ethylenediaminetetraacetic acid
G:F: Gain-to-Feed
Glu: L-Glutamate
H&E: Hematoxylin and Eosin
LPS: Lipopolysaccharide
LRT: Likelihood ratio test
MUC: Mucin
mTOR: Mammalian target of rapamycin
NC: Negative control
NF-κB: Nuclear factor kappa-light-chain-enhancer of activated B cells
NO: Nitric oxide
OCDN: Occludin
PBS: Phosphate-buffered saline
PC: Positive control
PCoA: Principal coordinate analysis
PCR: Polymerase chain reaction
PI: Post-inoculation
PWD: Post-weaning diarrhea
RT-qPCR: Reverse transcription quantitative polymerase chain reaction
RFLP: Restriction fragment length polymorphism
RNA: Ribonucleic acid
TCA: Tricarboxylic acid
VCR: Villus height-to-crypt depth ratio
ZO-1: Zonula occludens-1

## References

[1] Moeser AJ, Pohl CS, Rajput M. Weaning stress and gastrointestinal barrier development: Implications for lifelong gut health in pigs. Anim Nutr. 2017;3:313–21. 29767141 10.1016/j.aninu.2017.06.003 PMC5941262

[2] McCracken BA, Spurlock ME, Roos MA, Zuckermann FA, Gaskins HR. Weaning anorexia may contribute to local inflammation in the piglet small intestine. J Nutr. 1999;129:613–9. 10082764 10.1093/jn/129.3.613

[3] Heo JM, Opapeju FO, Pluske JR, Kim JC, Hampson DJ, Nyachoti CM. Gastrointestinal health and function in weaned pigs: a review of feeding strategies to control post-weaning diarrhoea without using in-feed antimicrobial compounds: Feeding strategies without using in-feed antibiotics. J Anim Physiol Anim Nutr. 2013;97:207–37. 10.1111/j.1439-0396.2012.01284.x 22416941

[4] Moeser AJ, Klok CV, Ryan KA, Wooten JG, Little D, Cook VL, et al. Stress signaling pathways activated by weaning mediate intestinal dysfunction in the pig. Am J Physiol-Gastrointest Liver Physiol. 2007;292:G173–81. 16901995 10.1152/ajpgi.00197.2006

[5] Overman EL, Rivier JE, Moeser AJ. CRF induces intestinal epithelial barrier injury via the release of mast cell proteases and TNF-α. PLoS ONE. 2012;7:e39935. 10.1371/journal.pone.0039935 PMC3386952 22768175

[6] Smith F, Clark JE, Overman BL, Tozel CC, Huang JH, Rivier JEF, et al. Early weaning stress impairs development of mucosal barrier function in the porcine intestine. Am J Physiol-Gastrointest Liver Physiol. 2010;298:G352–63. 19926814 10.1152/ajpgi.00081.2009 PMC2838512

[7] Gresse R, Chaucheyras-Durand F, Fleury MA, Van de Wiele T, Forano E, Blanquet-Diot S. Gut microbiota dysbiosis in postweaning piglets: Understanding the keys to health. Trends Microbiol. 2017;25:851–73. 28602521 10.1016/j.tim.2017.05.004

[8] Han X, Hu X, Jin W, Liu G. Dietary nutrition, intestinal microbiota dysbiosis and post-weaning diarrhea in piglets. Anim Nutr. 2024;17:188–207. 38800735 10.1016/j.aninu.2023.12.010 PMC11126776

[9] Monger XC, Gilbert A-A, Saucier L, Vincent AT. Antibiotic resistance: From pig to meat. Antibiotics. 2021;10:1209. 34680790 10.3390/antibiotics10101209 PMC8532907

[10] Wu G. Functional amino acids in nutrition and health. Amino Acids. 2013;45:407–11. 23595206 10.1007/s00726-013-1500-6

[11] Rezaei R, Knabe DA, Tekwe CD, Dahanayaka S, Ficken MD, Fielder SE, et al. Dietary supplementation with monosodium glutamate is safe and improves growth performance in postweaning pigs. Amino Acids. 2013;44:911–23. 23117836 10.1007/s00726-012-1420-x

[12] Duan J, Yin J, Ren W, Liu T, Cui Z, Huang X, et al. Dietary supplementation with l-glutamate and l-aspartate alleviates oxidative stress in weaned piglets challenged with hydrogen peroxide. Amino Acids. 2016;48:53–64. 26255283 10.1007/s00726-015-2065-3

[13] Hou Y, Wu G. L-Glutamate nutrition and metabolism in swine. Amino Acids. 2018;50:1497–510. 30116978 10.1007/s00726-018-2634-3

[14] Wang H, Liu Y, Shi H, Wang X, Zhu H, Pi D, et al. Aspartate attenuates intestinal injury and inhibits TLR4 and NODs/NF-κB and p38 signaling in weaned pigs after LPS challenge. Eur J Nutr. 2017;56:1433–43. 26907088 10.1007/s00394-016-1189-x

[15] Li Y, Han H, Yin J, He X, Tang Z, Li T, et al. D- and L-Aspartate regulates growth performance, inflammation and intestinal microbial community in young pigs. Food Funct. 2019;10:1028–37. 10.1039/c8fo01410h 30706916

[16] Liu G, Gu K, Liu X, Jia G, Zhao H, Chen X, et al. Dietary glutamate enhances intestinal immunity by modulating microbiota and Th17/Treg balance-related immune signaling in piglets after lipopolysaccharide challenge. Food Res Int. 2023;166:112597. 10.1016/j.foodres.2023.112597 36914323

[17] Rhouma M, Fairbrother JM, Beaudry F, Letellier A. Post weaning diarrhea in pigs: risk factors and non-colistin-based control strategies. Acta Vet Scand. 2017;59:31. 28526080 10.1186/s13028-017-0299-7 PMC5437690

[18] Zhang Y, Tan P, Zhao Y, Ma X. Enterotoxigenic *Escherichia coli* : intestinal pathogenesis mechanisms and colonization resistance by gut microbiota. Gut Microbes. 2022;14:2055943. 35358002 10.1080/19490976.2022.2055943 PMC8973357

[19] Tang Q, Lan T, Zhou C, Gao J, Wu L, Wei H, et al. Nutrition strategies to control post-weaning diarrhea of piglets: From the perspective of feeds. Anim Nutr. 2024;17:297–311. 38800731 10.1016/j.aninu.2024.03.006 PMC11127239

[20] Qin Q, Xu X, Wang X, Wu H, Zhu H, Hou Y, et al. Glutamate alleviates intestinal injury, maintains mTOR and suppresses TLR4 and NOD signaling pathways in weanling pigs challenged with lipopolysaccharide. Sci Rep. 2018;8:15124. 30310102 10.1038/s41598-018-33345-7 PMC6181909

[21] Li Y, Han H, Yin J, Zheng J, Zhu X, Li T, et al. Effects of glutamate and aspartate on growth performance, serum amino acids, and amino acid transporters in piglets. Food Agric Immunol. 2018;29:675–87.

[22] Pi D, Liu Y, Shi H, Li S, Odle J, Lin X, et al. Dietary supplementation of aspartate enhances intestinal integrity and energy status in weanling piglets after lipopolysaccharide challenge. J Nutr Biochem. 2014;25:456–62. 24565675 10.1016/j.jnutbio.2013.12.006

[23] National Research Council (U.S.). Nutrient requirements of swine. 11th rev. ed. Washington, D.C: National Academies Press; 2012.

[24] Kim K, He Y, Xiong X, Ehrlich A, Li X, Raybould H, et al. Dietary supplementation of *Bacillus subtilis* influenced intestinal health of weaned pigs experimentally infected with a pathogenic *E. coli*. J Anim Sci Biotechnol. 2019;10:52.

[25] Liu Y, Song M, Che TM, Almeida JAS, Lee JJ, Bravo D, et al. Dietary plant extracts alleviate diarrhea and alter immune responses of weaned pigs experimentally infected with a pathogenic *Escherichia coli*1. J Anim Sci. 2013;91:5294–306. 24045466 10.2527/jas.2012-6194

[26] DebRoy C, Maddox CW. Identification of virulence attributes of gastrointestinal *Escherichia coli* isolates of veterinary significance. Anim Health Res Rev. 2001;2:129–40. 11831435

[27] Rintarhat P, Cho Y-J, Koh H, Park S, Lee EJ, Lim H, et al. Assessment of DNA extraction methods for human gut mycobiome analysis. R Soc Open Sci. 2024;11:231129. 10.1098/rsos.231129 PMC10776226 38204788

[28] Caporaso JG, Lauber CL, Walters WA, Berg-Lyons D, Lozupone CA, Turnbaugh PJ, et al. Global patterns of 16S rRNA diversity at a depth of millions of sequences per sample. Proc Natl Acad Sci. 2011;108:4516–22. 20534432 10.1073/pnas.1000080107 PMC3063599

[29] Roehr JT, Dieterich C, Reinert K. Flexbar 3.0 – SIMD and multicore parallelization. Bioinformatics. 2017;33:2941–2. 10.1093/bioinformatics/btx330 28541403

[30] Callahan BJ, McMurdie PJ, Rosen MJ, Han AW, Johnson AJA, Holmes SP. DADA2: High-resolution sample inference from Illumina amplicon data. Nat Methods. 2016;13:581–3. 27214047 10.1038/nmeth.3869 PMC4927377

[31] Quast C, Pruesse E, Yilmaz P, Gerken J, Schweer T, Yarza P, et al. The SILVA ribosomal RNA gene database project: improved data processing and web-based tools. Nucleic Acids Res. 2012;41:D590–6. 23193283 10.1093/nar/gks1219 PMC3531112

[32] Tang X, Xiong K, Fang R, Li M. Weaning stress and intestinal health of piglets: A review. Front Immunol. 2022;13:1042778. 36505434 10.3389/fimmu.2022.1042778 PMC9730250

[33] Wu G. Amino acids: metabolism, functions, and nutrition. Amino Acids. 2009;37:1–17. 19301095 10.1007/s00726-009-0269-0

[34] Burrin DG, Stoll B. Metabolic fate and function of dietary glutamate in the gut. Am J Clin Nutr. 2009;90:850S-856S. 19587091 10.3945/ajcn.2009.27462Y

[35] Hou Y, Yao K, Wang L, Ding B, Fu D, Liu Y, et al. Effects of α-ketoglutarate on energy status in the intestinal mucosa of weaned piglets chronically challenged with lipopolysaccharide. Br J Nutr. 2011;106:357–63. 21342606 10.1017/S0007114511000249

[36] Kyoung H, Lee JJ, Cho JH, Choe J, Kang J, Lee H, et al. Dietary glutamic acid modulates immune responses and gut health of weaned pigs. Animals. 2021;11:504. 33671988 10.3390/ani11020504 PMC7919271

[37] Yin J, Liu M, Ren W, Duan J, Yang G, Zhao Y, et al. Effects of dietary supplementation with glutamate and aspartate on diquat-induced oxidative stress in piglets. PLOS ONE. 2015;10:e0122893. 10.1371/journal.pone.0122893 PMC4398417 25875335

[38] Zhou X, Liang J, Xiong X, Yin Y. Amino acids in piglet diarrhea: Effects, mechanisms and insights. Anim Nutr. 2024;16:267–74. 38362520 10.1016/j.aninu.2023.07.009 PMC10867606

[39] Wang H, Zheng X, Liu B, Xia Y, Xin Z, Deng B, et al. Aspartate metabolism facilitates IL-1β production in inflammatory macrophages. Front Immunol. 2021;12:753092. 10.3389/fimmu.2021.753092 PMC8567039 34745126

[40] Lee B, Moon KM, Kim CY. Tight junction in the intestinal epithelium: Its association with diseases and regulation by phytochemicals. J Immunol Res. 2018;2018:1–11. 10.1155/2018/2645465 PMC6311762 30648119

[41] Paradis T, Bègue H, Basmaciyan L, Dalle F, Bon F. Tight junctions as a key for pathogen invasion in intestinal epithelial cells. Int J Mol Sci. 2021;22:2506. 33801524 10.3390/ijms22052506 PMC7958858

[42] Wang L, Hu R, Ma S, Yang X, Gong J, Xiang H, et al. Dihydroquercetin attenuated Prevotella copri-caused intestinal injury by modulating gut microbiota and bile acids in weaned piglets. Anim Nutr. 2025;20:303–10. 39995524 10.1016/j.aninu.2024.10.002 PMC11849659

[43] Liao SF, Ji F, Fan P, Denryter K. Swine gastrointestinal microbiota and the effects of dietary amino acids on its composition and metabolism. Int J Mol Sci. 2024;25:1237. 38279233 10.3390/ijms25021237 PMC10816286

[44] Karasova D, Crhanova M, Babak V, Jerabek M, Brzobohaty L, Matesova Z, et al. Development of piglet gut microbiota at the time of weaning influences development of postweaning diarrhea – A field study. Res Vet Sci. 2021;135:59–65. 33444908 10.1016/j.rvsc.2020.12.022

[45] Guevarra RB, Hong SH, Cho JH, Kim B-R, Shin J, Lee JH, et al. The dynamics of the piglet gut microbiome during the weaning transition in association with health and nutrition. J Anim Sci Biotechnol. 2018;9:54. 30069307 10.1186/s40104-018-0269-6 PMC6065057

[46] Mahmud MR, Jian C, Uddin MK, Huhtinen M, Salonen A, Peltoniemi O, et al. Impact of intestinal microbiota on growth performance of suckling and weaned piglets. Microbiol Spectr. 2023;11:e03744–22. 10.1128/spectrum.03744-22 PMC10269657 37022154

[47] Rajilić-Stojanović M, De Vos WM. The first 1000 cultured species of the human gastrointestinal microbiota. FEMS Microbiol Rev. 2014;38:996–1047. 24861948 10.1111/1574-6976.12075 PMC4262072

[48] Wu G, Bazer FW, Davis TA, Jaeger LA, Johnson GA, Kim SW, et al. Important roles for the arginine family of amino acids in swine nutrition and production. Livest Sci. 2007;112:8–22.

[49] Bin P, Liu S, Chen S, Zeng Z, Huang R, Yin Y, et al. The effect of aspartate supplementation on the microbial composition and innate immunity on mice. Amino Acids. 2017;49:2045–51. 28733903 10.1007/s00726-017-2467-5

[50] Deters BJ, Saleem M. The role of glutamine in supporting gut health and neuropsychiatric factors. Food Sci Hum Wellness. 2021;10:149–54.

