# Supplementary material for: Effects of dietary L-glutamate and L-aspartate supplementation on growth performance, severity of diarrhea, intestinal barrier integrity, and fecal microbiota of weaned piglets challenged with F18 enterotoxigenic Escherichia coli
Source: J Anim Sci Biotechnol. 2025 Sep 30;16:131. doi: 10.1186/s40104-025-01266-x (PMC12482100; doi:10.1186/s40104-025-01266-x)
Supplement: Supplementary file 1 — Supplementary Material 1. Table S1 Amino acid profiles of experimental diets. Table S2 Sequences of oligonucleotide primers used for RT-qPCR assay. Fig. S1 Bacterial phyla in pig gut. A Phylum level microbial distribution in pig gut. B Significant differences between phyla proportions were found at different timepoints. Fig. 2 Alpha diversity differences between different treatment groups on d −7 (A) and d 0 (B) are not significant. Fig. S3 Distribution of 20 most abundant genera in pigs in different treatment groups. Fig. 4 Significant differences in taxa proportions were found between treatment groups on d 14 post-inoculation. [file 40104_2025_1266_MOESM1_ESM.docx]

**Table S1** Amino acid profiles of experimental diets

| **Item^1^** | **NC** | **PC** | **PC+1% Glu** | **PC+2%Glu** | **PC+1%Asp** | **PC+2%Asp** | **PC+carbadox** |
| --- | --- | --- | --- | --- | --- | --- | --- |
| Phase I diet |  |  |  |  |  |  |  |
| Arginine | 1.18 | 1.28 | 1.29 | 1.21 | 1.24 | 1.13 | 1.24 |
| Histidine | 0.48 | 0.52 | 0.52 | 0.49 | 0.50 | 0.46 | 0.50 |
| Isoleucine | 0.94 | 1.01 | 1.01 | 0.96 | 0.98 | 0.90 | 0.99 |
| Leucine | 1.64 | 1.74 | 1.72 | 1.63 | 1.68 | 1.56 | 1.70 |
| Lysine | 1.43 | 1.52 | 1.48 | 1.51 | 1.46 | 1.37 | 1.47 |
| Methionine | 0.46 | 0.48 | 0.44 | 0.46 | 0.41 | 0.40 | 0.46 |
| Phenylalanine | 0.91 | 0.97 | 0.98 | 0.92 | 0.95 | 0.87 | 0.95 |
| Threonine | 0.83 | 0.89 | 0.88 | 0.84 | 0.83 | 0.80 | 0.85 |
| Tryptophan | 0.24 | 0.24 | 0.24 | 0.23 | 0.22 | 0.22 | 0.24 |
| Valine | 0.98 | 1.05 | 1.05 | 0.99 | 1.01 | 0.94 | 1.02 |
| Alanine | 0.98 | 1.03 | 1.01 | 0.96 | 1.01 | 0.92 | 1.00 |
| Aspartic acid | 1.93 | 2.11 | 2.13 | 2.00 | 2.98 | 3.87 | 2.04 |
| Cysteine | 0.30 | 0.32 | 0.32 | 0.30 | 0.30 | 0.28 | 0.31 |
| Glutamic acid | 3.35 | 3.60 | 4.47 | 5.52 | 3.53 | 3.25 | 3.53 |
| Glycine | 0.84 | 0.91 | 0.89 | 0.84 | 0.89 | 0.80 | 0.87 |
| Proline | 1.01 | 1.07 | 1.06 | 1.00 | 1.05 | 0.97 | 1.04 |
| Serine | 0.77 | 0.83 | 0.83 | 0.78 | 0.81 | 0.75 | 0.82 |
| Tyrosine | 0.59 | 0.63 | 0.64 | 0.61 | 0.63 | 0.58 | 0.63 |
| Phase II diet |  |  |  |  |  |  |  |
| Arginine | 1.25 | 1.23 | 1.21 | 1.17 | 1.27 | 1.20 | 1.24 |
| Histidine | 0.50 | 0.50 | 0.49 | 0.48 | 0.52 | 0.49 | 0.50 |
| Isoleucine | 0.94 | 0.94 | 0.92 | 0.90 | 0.98 | 0.91 | 0.95 |
| Leucine | 1.66 | 1.66 | 1.63 | 1.58 | 1.71 | 1.60 | 1.66 |
| Lysine | 1.41 | 1.41 | 1.38 | 1.39 | 1.44 | 1.43 | 1.49 |
| Methionine | 0.43 | 0.39 | 0.39 | 0.38 | 0.38 | 0.41 | 0.42 |
| Phenylalanine | 0.95 | 0.94 | 0.93 | 0.90 | 0.98 | 0.92 | 0.95 |
| Threonine | 0.83 | 0.83 | 0.78 | 0.81 | 0.81 | 0.80 | 0.81 |
| Tryptophan | 0.22 | 0.23 | 0.23 | 0.23 | 0.24 | 0.23 | 0.22 |
| Valine | 0.98 | 0.97 | 0.96 | 0.94 | 1.01 | 0.96 | 0.98 |
| Alanine | 0.94 | 0.94 | 0.93 | 0.90 | 0.96 | 0.92 | 0.94 |
| Aspartic acid | 1.99 | 1.97 | 1.94 | 1.91 | 2.98 | 3.79 | 2.00 |
| Cysteine | 0.31 | 0.30 | 0.32 | 0.29 | 0.32 | 0.29 | 0.33 |
| Glutamic acid | 3.51 | 3.51 | 4.28 | 5.29 | 3.60 | 3.40 | 3.53 |
| Glycine | 0.78 | 0.78 | 0.77 | 0.75 | 0.81 | 0.77 | 0.79 |
| Proline | 1.03 | 1.03 | 1.01 | 0.98 | 1.06 | 1.00 | 1.03 |
| Serine | 0.82 | 0.80 | 0.79 | 0.77 | 0.84 | 0.78 | 0.81 |
| Tyrosine | 0.62 | 0.61 | 0.60 | 0.59 | 0.64 | 0.61 | 0.62 |

^1^W/W% = grams per 100 grams of sample. Results are expressed on an "as is" basis unless otherwise indicated

**Table S2** Sequences of oligonucleotide primers used for RT-qPCR assay

| **Gene^1^** | **Primer sequence (5’-3’)^2^** | **Ta (°C)^3^** | **Acc. No.^4^** |  |
| --- | --- | --- | --- | --- |
| *18S rRNA* | F: AGGAAAGCAGACATCGACCT  R: ACCTGGCTGTACTTCCCATC | 59.23 | AB117609.1 | |
| *MUC-2* | F: AAGGACGACACCATCTACCTCACT  R: GGCCAGCTCGGGAATAGAC | 61.06 | XM_021082584.1 | |
| *ZO-1* | F: CCGCCTCCTGAGTTTGATAG  R: CAGCTTTAGGCACTGTGCTG | 57.21 | AJ318101 | |
| *CLDN-1* | F: ACTGGCTGGGCTGCTGCTTCTCT  R: GGATAGGGCCTTGGTGTTGGGTAA | 55 | NM_001244539.1 | |
| *OCDN* | F: TTCATTGCTGCATTGGTGAT  R: ACCATCACACCCAGGATAGC | 54.6 | NM_001163647.2 | |

*^1^18S rRNA* = 18S ribosomal ribonucleic acid; *MUC-2* = mucin-2; *ZO-1* = zonula occludens-1; *CLDN-1* = claudin-1; *OCLN* = occludin

^2^F = forward; R = reverse

^3^Ta = annealing temperature

^4^Acc. No. = accession number in GenBank database


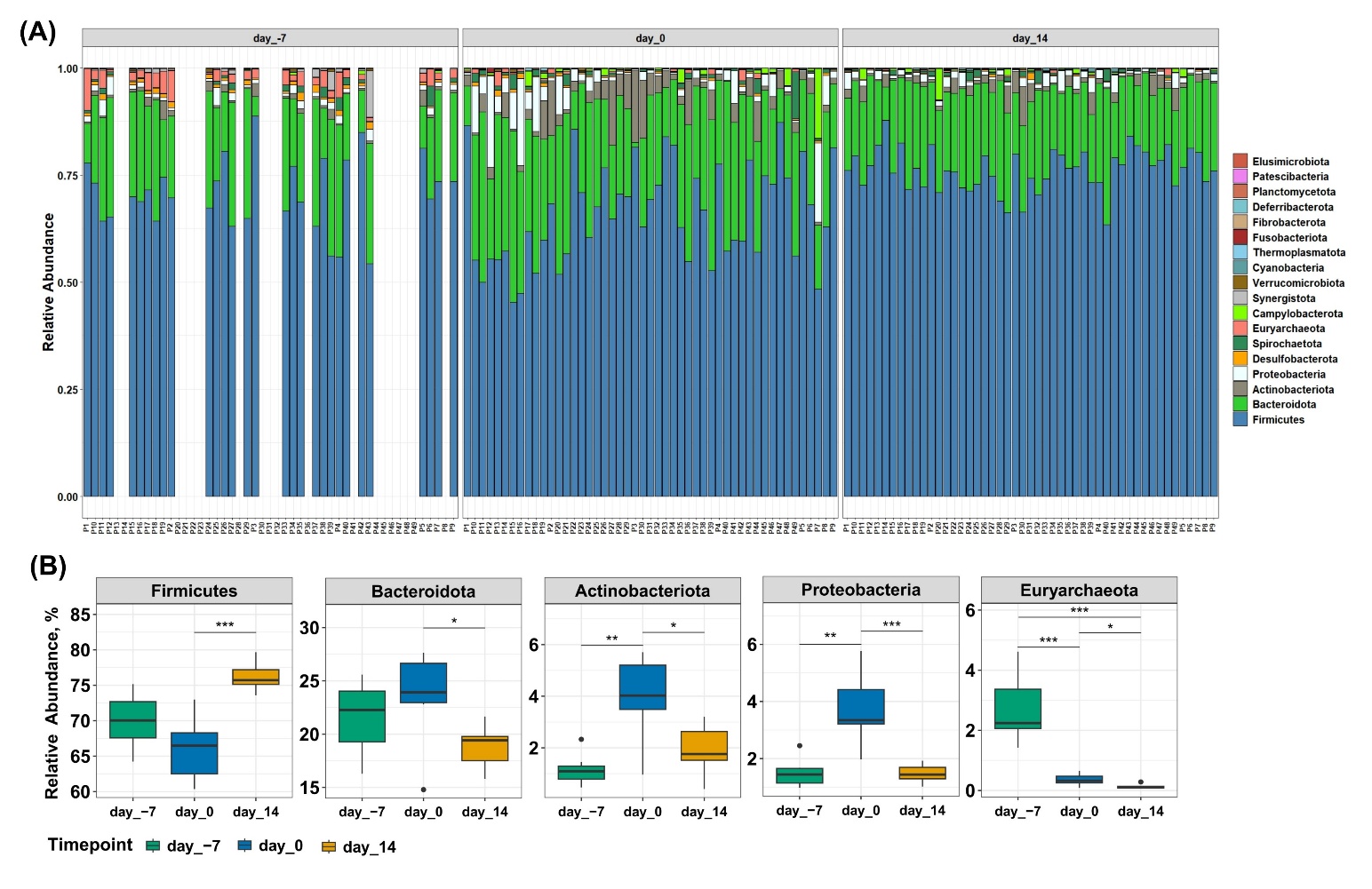


**Fig. S1** Bacterial phyla in pig gut. **A** Phylum level microbial distribution in pig gut. *X*-axis shows individual pigs faceted by sampling timepoints. **B** Significant differences between phyla proportions were found at different timepoints. Significant differences are indicated by the adjusted *P*-value (* < 0.05, ** < 0.01, *** < 0.005)


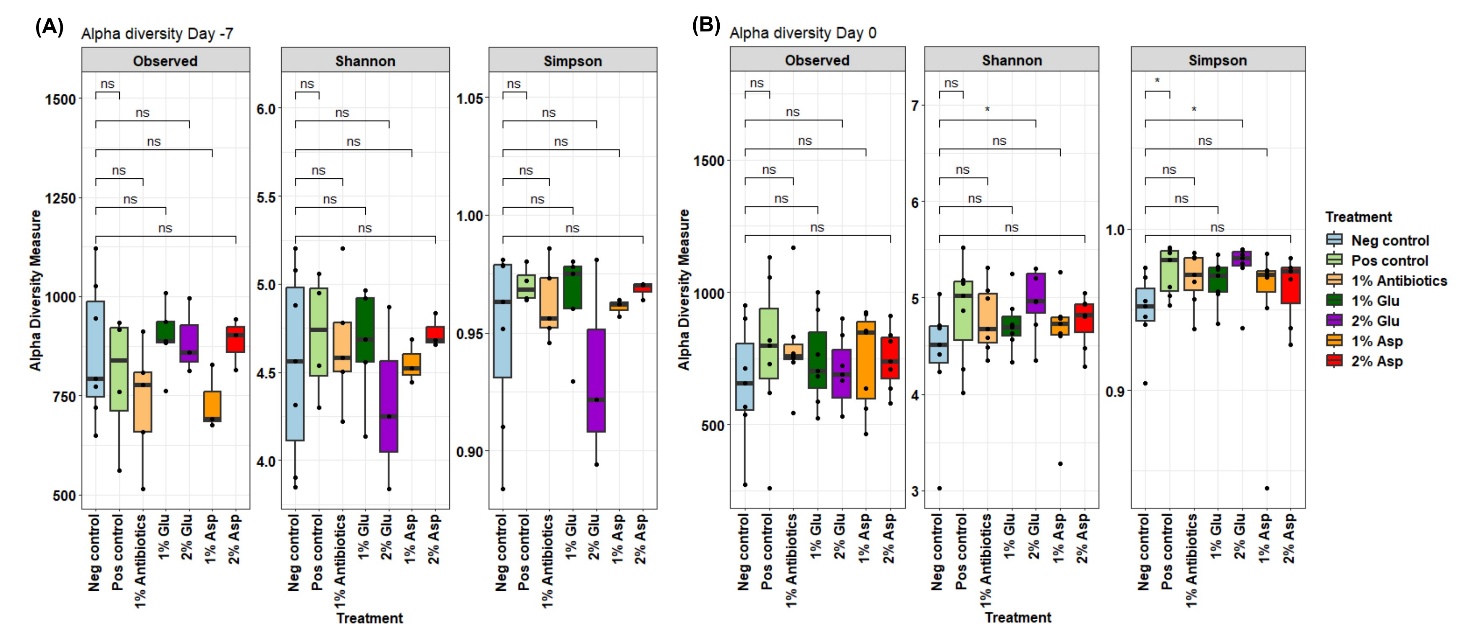


**Fig. 2** Alpha diversity differences between different treatment groups on d −7 and d 0 are not significant. Alpha diversity matrices (Observed, Shannon, Simpson) were calculated on sampling timepoint d −7 (**A**), and d 0 (**B**) for pigs treated with dietary supplements (*X*-axis). Significant differences between the groups were marked based on the adjusted *P*-value (* < 0.05)


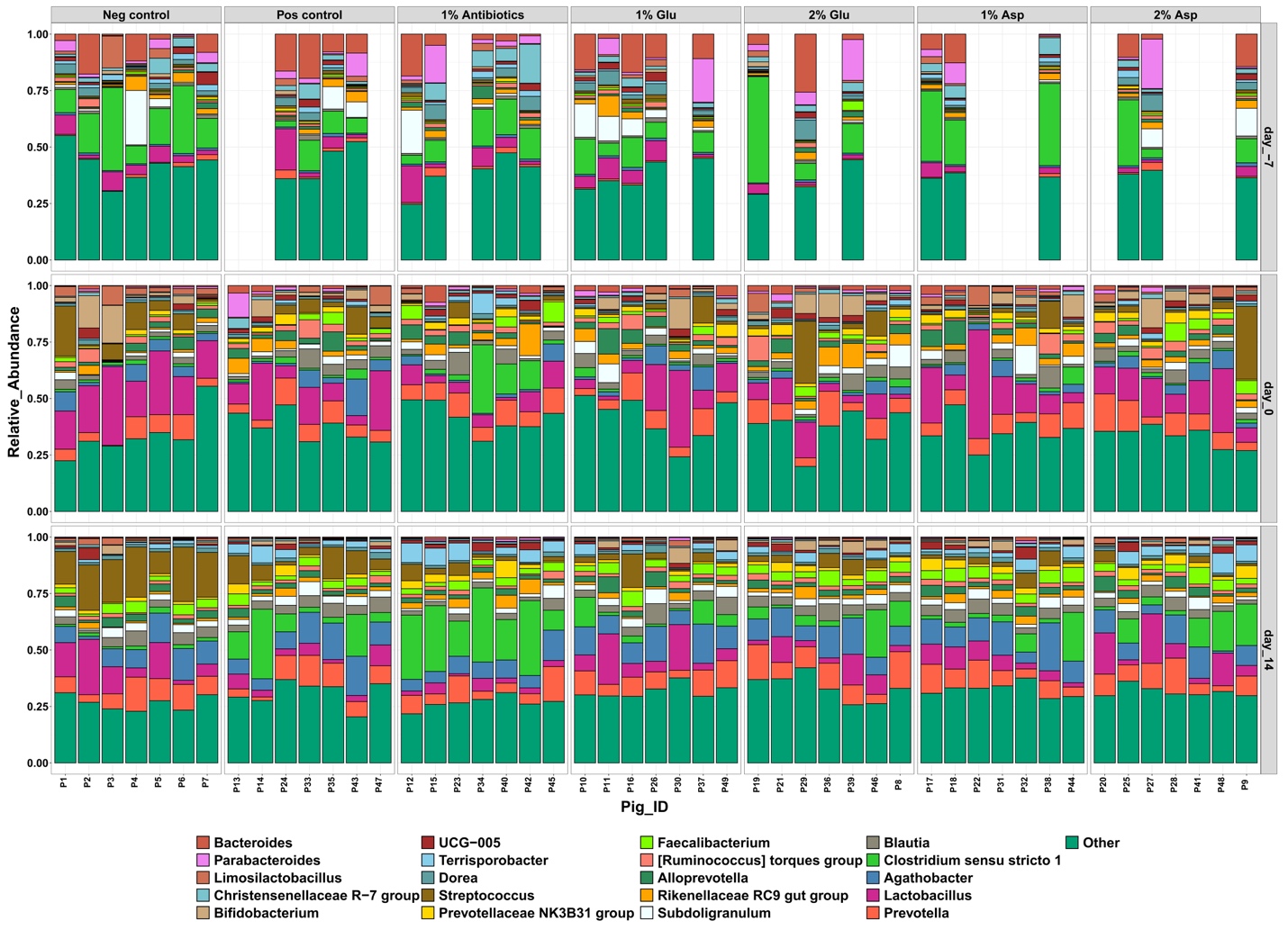


**Fig. S3** Distribution of 20 most abundant genera in pigs in different treatment groups. *X*-axis shows individual pigs faceted by treatment groups. *Y*-axis depicts the relative abundance of the most abundant genera at d −7, d 0 and d 14 post-inoculation. Empty columns at d −7 indicate the absence of a fecal sample


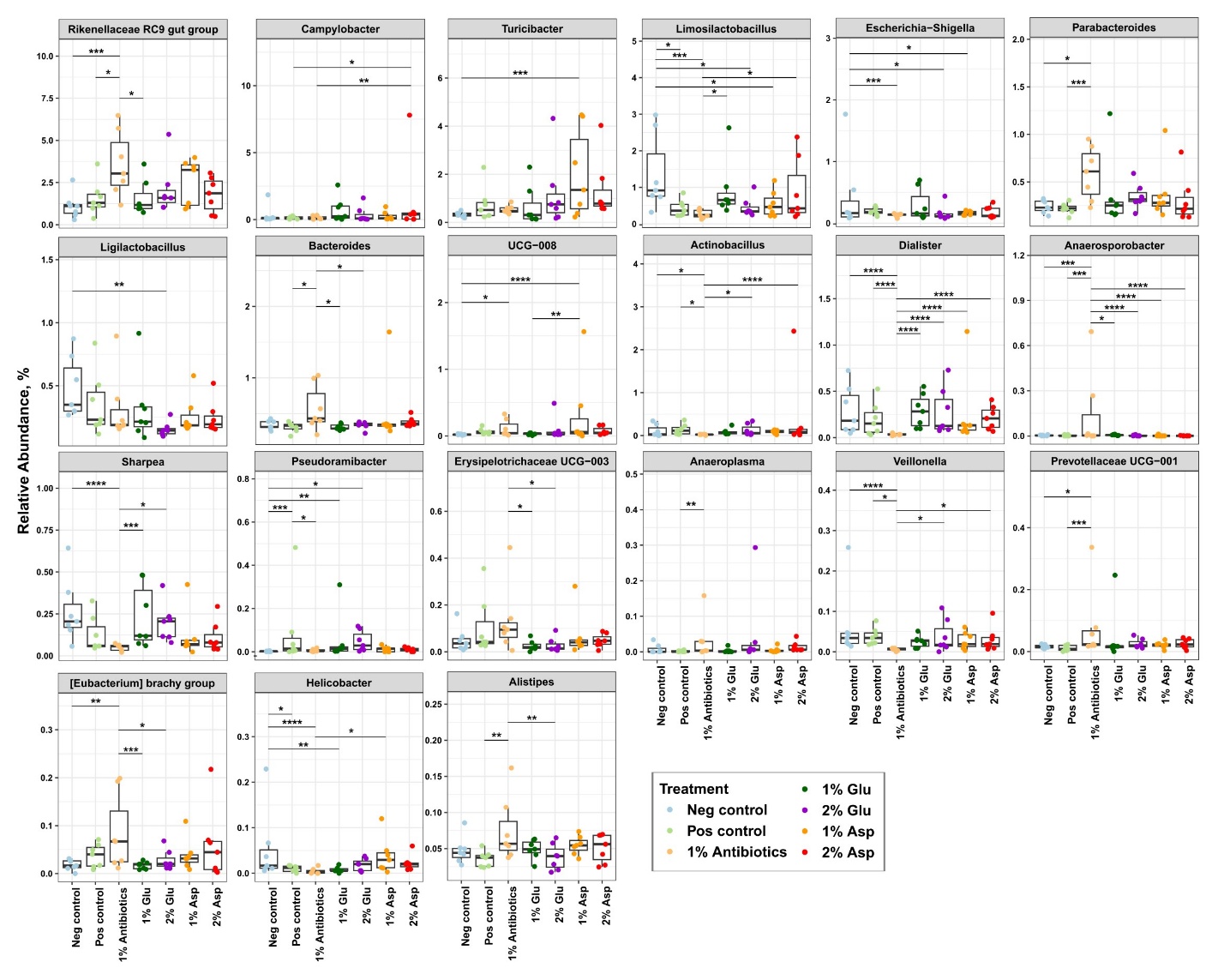


**Fig. 4** Significant differences in taxa proportions were found between treatment groups on d 14 post-inoculation. Significant differences between treatment groups are indicated by the adjusted *P*-value (* < 0.05, ** < 0.01, *** < 0.005, **** < 0.001)
